# Is the intimal thickness a key contributor to thoracic aortopathy?

**DOI:** 10.1007/s12055-023-01528-1

**Published:** 2023-06-05

**Authors:** Nimrat Grewal, Robert Poelmann

**Affiliations:** 1 Department of Cardiothoracic Surgery, Amsterdam University Medical Center, Amsterdam, the Netherlands; 2 Department of Cardiothoracic Surgery, Leiden University Medical Center, Leiden, the Netherlands; 3 Department of Anatomy and Embryology, Leiden University Medical Center, Leiden, the Netherlands; 4 Institute of Biology, Animal Sciences and Health, Leiden University, Leiden, the Netherlands; 5 Department of Cardiology, Leiden University Medical Center, Leiden, the Netherlands

**Keywords:** Bicuspid aortic valve, Marfan syndrome, Type A dissection, Pathology, Aortopathy

## Abstract

**Background:**

An aortic dissection is the most devastating complication of thoracic aortic disease. Several non- and syndromic conditions such as a bicuspid aortic valve (BAV) and Marfan syndrome (MFS) have a severely increased risk to develop a thoracic aortic aneurysm and dissection. To date, the medial layer has been extensively studied in search of the pathogenetic mechanisms leading to aortic complications.

**Objective:**

We aim to determine whether intimal layer pathology is characteristic in all thoracic aortopathy regardless of the underlying etiology.

**Method:**

A total of 176 aortic wall specimen were studied for the intimal layer architecture including the intimal thickness, endothelial cell morphology, and atherosclerosis. Specimens were derived from four patient groups: BAV (*n* = 70, age 57 ± 8.9 years), isolated tricuspid aortic valve (TAV) (*n* = 38, age 64.9 ± 11.0 years), MFS with a TAV (*n* = 8, age 34.2 ± 11.0 years), type A dissections with a TAV (*n* = 60, age 62.7 ± 10 years).

**Results:**

The intimal layer is significantly thinner in BAV, MFS, and type A aortic dissection as compared to the isolated TAV patients (*p* < 0.001). Intimal atherosclerosis was also significantly less present in the three groups as compared to the isolated TAV (*p* < 0.05).

**Discussion:**

A thin intimal layer is a common finding in the thoracic aortopathy patients. Studies aiming at preventing future aortic complications should focus on the intimal pathology as a common effector pathway in thoracic aortopathy.

## Introduction

A bicuspid aortic valve (BAV) is the most common congenital cardiac anomaly and Marfan syndrome (MFS) is one of the most common connective tissue disorders. Both conditions share many clinical and pathophysiological properties, such as a high risk for thoracic aortic aneurysm development and dissections (TAADs), with serious morbidity and mortality rates. Currently, TAADs can only be managed by prophylactic aortic surgery of larger aneurysms (4.5–5 cm in diameter) when individuals at risk are identified. Although blood pressure–lowering drugs limit aneurysm growth somewhat before reaching this threshold, no drugs have been found so far that can halt aneurysm growth to prevent aortic events such as dissection or rupture. Consequently, pharmaceutical stabilization of aortic aneurysms is considered an urgent medical need. Most focus for drug discovery has been on the aortic smooth muscle cells as TAADs in BAV and MFS associate with changing smooth muscle cell phenotype and smooth muscle cell death. Our previous studies have shown that in BAV individuals, besides the smooth muscle cell defects, the intimal layer is also structurally different as compared to their tricuspid aortic valve counterparts [1, 2]. Intimal components, such as endothelial cells, communicate with smooth muscle cells and are in contact with the circulation, promising an easy drug target.

The aim of this study is to investigate the structure of the intimal layer in BAV, TAV, MFS, and type A aortic dissection patients and to determine whether intimal layer pathology is characteristic in all thoracic aortopathy regardless of the underlying etiology.

We hypothesize that an abnormally developed intimal layer increases susceptibility for future aortic complications. In this paper, we describe the intimal architecture in non- and syndromic thoracic aortopathy.

## Material and methods

### Ascending aortic wall specimen

Non- and dilated ascending aortic wall tissue samples were collected from individuals with BAV and a tricuspid aortic valve (TAV). A non-dilated aorta was clinically defined by an ascending aortic wall diameter of  < 45 mm, based on the cutoff for concomitant aortic surgery in the current guidelines [3]. A total of 176 patients were included in this study. Inclusion criteria were an age of 18 years or above. Patients were excluded if they were known with a genetic or connective tissue disorder other than MFS. Patients included in the study were operated between 2012 and 2020.

A total of 38 non-dilated (mean age 56.8 ± 9 years, 71% male) and 32 dilated BAV patients were studied (mean age 57.2 ± 9 years, 81% male). Material from BAV patients was available from patients undergoing an elective aortic valve or root replacement between 2013 and 2015 at the Leiden University Medical Center (LUMC), Leiden, the Netherlands, and the Central Hospital, Bad Berka, Germany. The Heart Valve Bank, Thoraxcenter, Erasmus Medical Center, Rotterdam, also provided six BAV samples without aortic dilatation as these were not suitable for transplantation, as approved by their Scientific Advisory Board. All BAV patients had a raphe between the right and left coronary cusp.

A total of 17 non-dilated TAV patients (mean age 63.2 ± 8 years, 65% male) were included, which were obtained during an elective aortic valve replacement at the Central Hospital and post-mortem from the LUMC. Twenty-one dilated TAV patients (mean age 66.3 ± 13 years, 52% male) were obtained during aortic root or ascending aortic replacement between 2013 and 2015 at the LUMC and at the Central Hospital. Additional TAV samples were obtained from patients with proven thoracic aortopathy: eight MFS patients (mean age 34.2 ± 11.0 years, 63% male) from the Amsterdam University Medical Center (AUMC), Amsterdam, and 60 type A aortic dissections (mean age 62.7 ± 10 years, 53% male) from the LUMC. All aortic samples were uniformly obtained from the aortotomy incision. In patients with replacement of the ascending aorta, the samples were approximately 1.0–1.5 cm^2^. In cases of an isolated aortic valve surgery, the specimen was approximately 0.3–0.5 cm^2^. Our previous study concerning the role of shear stress on the intimal layer did not show any difference between stenotic and regurgitant bicuspid and TAV pathology [4]; therefore, we did not differentiate in both pathologies in this paper.

### Ethics

Our study complies with the Declaration of Helsinki. For this study sample, collection and handling of aortic tissue were carried out according to the official guidelines of the Medical Ethical Committee of the LUMC, the AUMC, and the Central Hospital and approval for this study was granted by the Medial Ethical (reference number B21.051/MS/ms). All patients gave written informed consent.

All control specimens were obtained post-mortem. Obduction was performed according to the guidelines of the pathology department. Tissue collection was performed according to the regulations and protocols for secondary tissue use of the dept. of pathology at the LUMC.

### Routine histology, immunohistochemistry, and histopathological parameters

Transverse sections were stained with hematoxylin-eosin (5 μm), resorcin-fuchsin (5 μm), and Movat pentachrome staining (4 μm). Additional platelet endothelial cell adhesion molecule (PECAM) staining (5-μm section) was performed to visualize the endothelial cells; for the staining protocol, we refer to our previous publication [5].

Intimal architecture was studied, including endothelial cell morphology, the subendothelial layer structure, and the absolute intimal thickness in micrometers. The intima is defined as the area between the inner surface of the aortic wall, lined by the sometimes still present endothelial cells and the well-structured elastic lamellae of the media (excluding atherosclerotic areas).

Intimal atherosclerosis was studied and indexed from 0 (none), 1 (mild), 2 (moderate) to 3 (severe). All specimens were evaluated by two independent researchers, who were blinded to the collection site of aortic specimens.

### Statistical analysis

Numerical data are presented as mean ± standard deviation. Statistical differences were evaluated with the Mann-Whitney *U* test for comparison between the groups. Significance was assumed when *p* < 0.05 using the SPSS 25.0 software program. GraphPad software was used to create graphics of the statistical analyses.

## Results

**Fig. 1:**
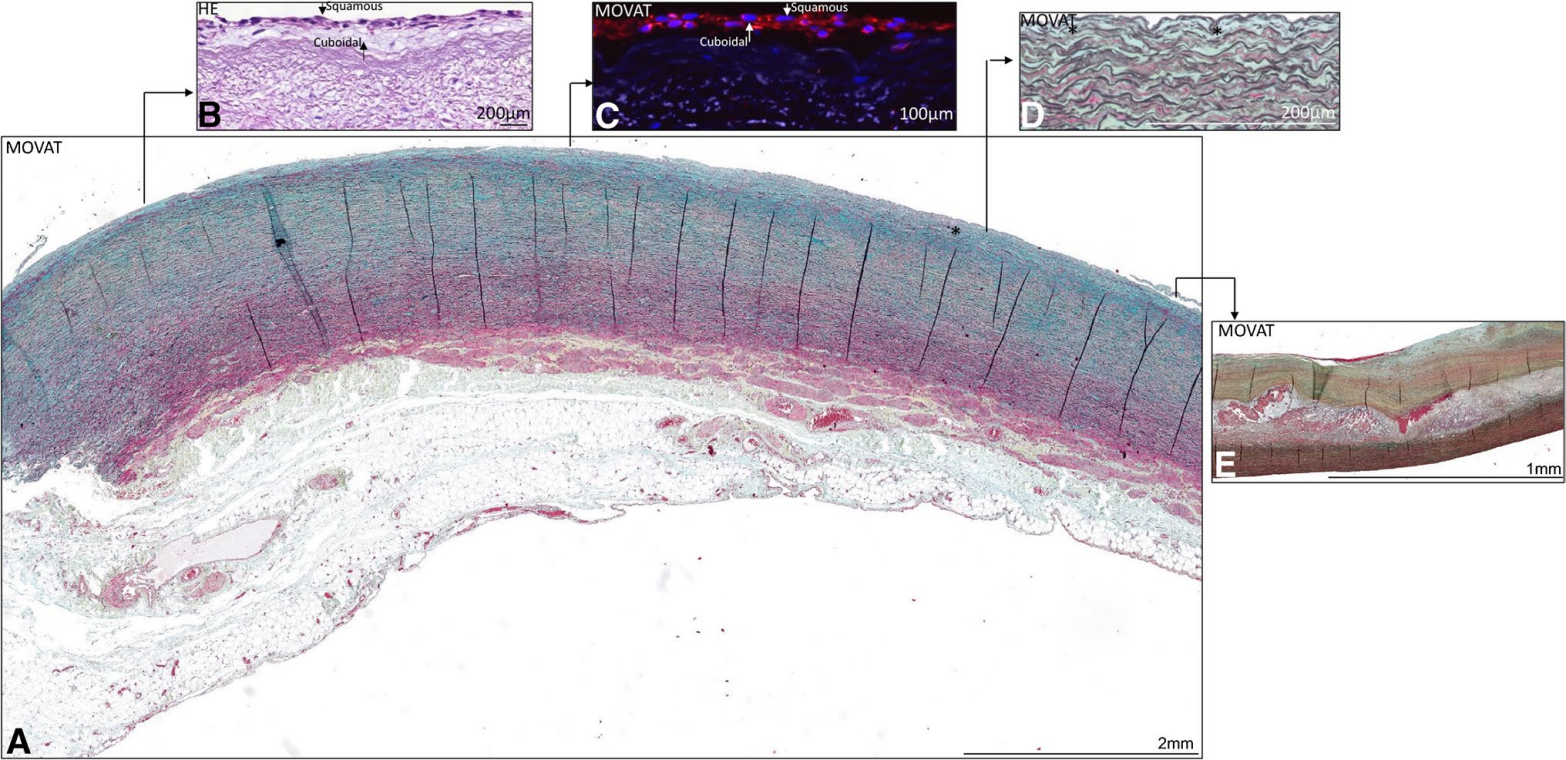
Transverse histologic sections of non-dilated ascending aortic specimen in two tricuspid aortic valve patients, 4 μm (**A**, **D**, **E**) and 5 μm (**B**, **C**). Stained with Movat pentachrome staining (**A**, **D**, **E**), hematoxylin-eosin (**B**), and PECAM (**C**). An overview of the ascending aortic wall is presented in **A**. **B**–**E** Obtained from a different patient and illustrate the features in detail. The endothelial cells are highlighted in **B** and **C**; varying cuboidal and squamous cells are depicted with an arrow. The subendothelial layer is indicated with an asterisk in **A** and **D** and atherosclerosis in **E**. Abbreviations: HE, hematoxylin-eosin

Endothelial cells were scarcely present in the aortic wall, due to mechanical damage of the inner layer of the specimen during tissue handling on the operation theatre. The endothelial cells which could be investigated in the study groups had a similar morphological appearance. In the BAV, TAV, MFS, and type A aortic dissection groups, the cells predominantly had a squamous morphology with a few cuboidal cells (Fig. 1A–C). The subendothelial layer consisted of fine elastic lamellae, with a few smooth muscle cells. In the TAV groups, the subendothelial layer was filled with mucoid extracellular matrix between the elastic lamellae (Fig. 1D). The BAV, MFS, and type A aortic dissection groups demonstrated a few fine elastic lamellae with hardly any mucoid extracellular matrix in between.

Intimal atherosclerosis was significantly less apparent in the BAV, MFS, and type A aortic dissection as compared to the TAV patients (*p* < 0.05) (Fig. 1E). Intimal thickness was defined as the area between the inner surface of the aortic wall, lined by the endothelial cells and the well-structured elastic lamellae of the media, excluding atherosclerotic areas if present.

**Fig. 2:**
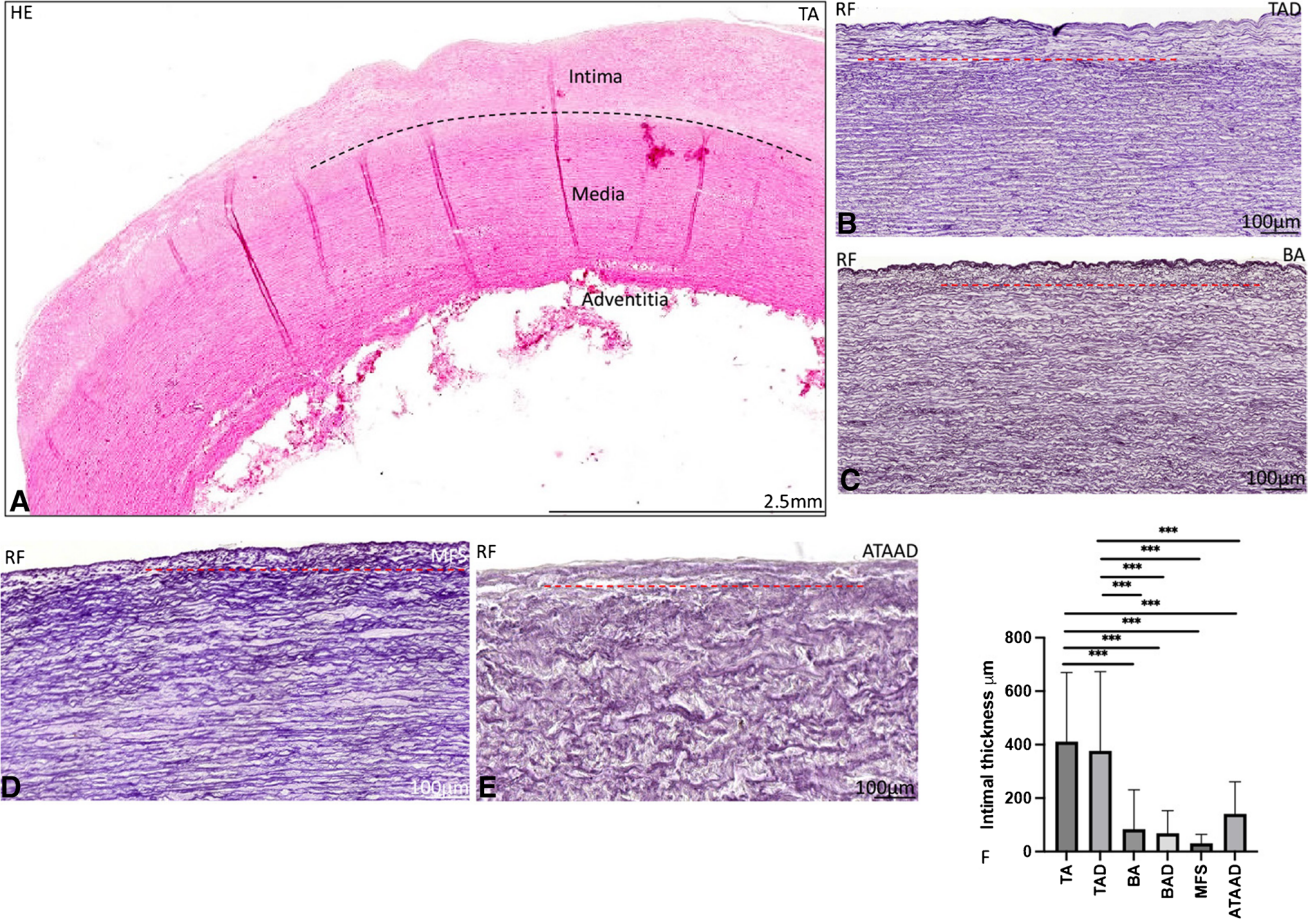
Transverse histologic sections of ascending aortic specimen (5 μm). Stained with hematoxylin–eosin (HE) (**A**) and resorcin-fuchsin (RF) (**B**–**E**). An overview of the ascending aortic wall in the non-dilated tricuspid aortic valve (TAV) is presented **A**, with a black dashed line depicting the border of the intimal layer. **B** Dilated tricuspid aortic valve with a red dashed line highlighting the intimal border. The intimal layer in the non-dilated BAV (**C**), MFS (**D**), and acute type A aortic dissection (**E**) is significantly thinner as compared to the isolated TAV patients (graph F). Abbreviations: HE, hematoxylin-eosin; RF, resorcin-fuchsin; TA, non-dilated tricuspid aortic valve; TAD, dilated tricuspid aortic valve; BA, non-dilated bicuspid aortic valve; MFS, Marfan syndrome; ATAAD, acute type A aortic dissection

All patients with a non- and dilated BAV, MFS, and type A aortic dissection had a significantly thinner intimal layer as compared to all non- and dilated TAV patients (*p* < 0.001)) (Fig. 2A–E, graph F).

No significant difference in intimal layer thickness was seen between the non- and dilated BAV, MFS, and type A aortic dissection group (graph F). No difference in intimal layer thickness was observed between the non- and dilated TAV groups (graph 2F).

Observed intimal differences in BAV, MFS, and type A aortic dissections were not found related to age or gender and are summarized in Table 1.

**Table 1:** Observed intimal differences in BAV, MFS, and type A aortic dissections

	BAV patients	MFS patients	TAAD patients	Isolated TAV patients
Endothelial cells	Squamous morphology with few cuboidal cells	Squamous morphology with few cuboidal cells	Squamous morphology with few cuboidal cells	Squamous morphology with few cuboidal cells
Atherosclerosis	Significantly less atherosclerosis compared to control patients (*p* < 0.05)	Significantly less atherosclerosis compared to control patients (*p* < 0.05)	Significantly less atherosclerosis compared to control patients (*p* < 0.05)	Significantly more atherosclerosis compared to the BAV, MFS, and TAAD patients (*p* < 0.05)
Intimal thickness	Significantly thinner intimal layer compared to control patients (*p* < 0.001)	Significantly thinner intimal layer compared to control patients (*p* < 0.001)	Significantly thinner intimal layer compared to control patients (*p* < 0.001)	Significantly thicker intimal layer compared to the BAV, MFS, and TAAD patients (*p* < 0.001)
MEMA in subendothelial layer	Less MEMA compared to control patients	Less MEMA compared to control patients	Less MEMA compared to control patients	More MEMA compared to control patients

Abbreviations: *BAV* bicuspid aortic valve, *MEMA* mucoid extracellular matrix accumulation, *MFS* Marfan syndrome, *TAAD* type A aortic dissection, *TAV* tricuspid aortic valve

## Discussion

A BAV is the most common cardiac anomaly characterized by an aortic valve with two instead of the normal three cusps (prevalence 1–2%). MFS is one of the most common connective tissue disorders, caused by mutations in the extracellular matrix gene Fibrillin-1 (FBN1) (incidence 1:5000). Both conditions associate with an increased risk for aortic aneurysm formation. Although generally asymptomatic, it can result in life-threatening complications such as aorta dissection and rupture. Due to lack of aorta growth-inhibiting medication, patients must be monitored regularly to determine if and when aortic surgery is necessary.

In both conditions of BAV and Marfan, smooth muscle cell differentiation defects are observed [6] and so far, most drug discovery studies have focused on the smooth muscle cells pathology. Less is known about the intimal pathology in non-and syndromic aortopathy, even though a type A aortic dissection is initiated by a tear in the intimal layer. Moreover, endothelial cells line the luminal surface and are in contact with the blood and communicate signals to the intimal and smooth muscle cell layer following the assumption that the intima is not a mere permissive passive layer. Therefore, the innermost layer of the aortic wall is interesting to study as a target for aortic disease treatment. This study aims at describing the intimal architecture in non- and syndromic aortopathy being BAV, MFS, and type A aortic dissection patients.

A recent study revealed a difference in the timing of intimal development in BAV and TAV patients [7]. In TAV, the expansion of intimal development is apparent neonatally (4 months), and the subendothelial layer thereafter increases in thickness until the age of six, after which the intimal layer stabilizes for a few years and again increases in thickness during adulthood [7]. In TAV, the increase in intimal thickness is regarded as a normal physiological process, important for strengthening of the vessel wall and can be seen as a remarkable ability to adapt to injury and/or regeneration and cope with high and pulsatile blood pressure. In the BAV, the intimal thickening starts in early gestation demonstrating an intimal thickness comparable to early life in TAV. Strikingly, after birth, the intima decreases in thickness to remain as a significantly thinner layer throughout life.

In the current study, we confirmed the findings that the adult BAV has a significantly thinner intima as compared to the patients with isolated TAV. Furthermore, despite having a morphological TAV, the MFS patients and type A aortic dissection patients also showed a significantly thinner intimal layer as compared to the isolated TAV groups. The latter emphasizes that thoracic aortopathy is the predominant factor associated with a thinner intimal layer, rather than the aortic valve morphology. We hypothesize that a common underlying pathology in the early developmental phase is responsible for the observed thinner intima in the various thoracic aortopathy conditions, leading to a lack in normal protective intimal proliferation. Endothelial cells lining the intimal layer play a crucial role in the development of the intimal layer by undergoing a transition to mesenchymal cells which is called endothelial to mesenchymal transition (EndoMT) [8]. Transforming growth factor beta (TGF-β) is an important factor in vessel wall maintenance and pathology. MFS is characterized by TGF-β dysregulation [9]. Among the known BAV hereditary genes, there are a number involved in TGF-β signaling as well (FBN1, TGFBR2, TGFB2, SMAD6) [10]. TGF-β is involved in EndoMT and is a potent activator of endothelial cells and enhances intimal hyperplasia through the production of extracellular matrix proteins while it suppresses smooth muscle cell activation [11]. Furthermore, TGF-β is critical for cardiac valve formation and smooth muscle differentiation. It thus seems obvious that TGF-β plays a central role in the intimal pathology and smooth muscle cell differentiation defects in non- and syndromic thoracic aortopathy.

Findings of the current descriptive study should therefore lay the foundation for further investigations to unravel the mechanisms and the genetic pathways involved in formation of the intima as a marker to distinguish patients with an increased risk for future aortopathy.

## Conclusion

The ascending aortic wall in non-and syndromic thoracic aortopathy patients is characterized by a significantly thinner intimal layer as compared to control patients. Considering the crucial role of normal intimal proliferation in the protection of the vascular wall, it is plausible that the abnormally developed intimal layer increases susceptibility for aortic complications in BAV, MFS, and TAAD patients.

### Limitations

We designed our study by comparing histopathological features in the ascending aorta of BAV, TAV, MFS, and type A dissection patients. A limitation of our semi-quantitative study approach is that we did not have frozen tissue samples of all the aortic wall specimens. Therefore, quantitative evaluation of described features to correlate our findings to immunohistochemistry could not be performed.

## Data Availability

The raw data supporting the conclusions of this article will be made available by the authors, without undue reservation.

## References

[1] Grewal N, Girdauskas E, Idhrees M, Velayudhan B, Klautz R, Driessen A (2023). Structural abnormalities in the non-dilated ascending aortic wall of bicuspid aortic valve patients. Cardiovasc Pathol.

[2] Grewal N, Groot ACG-d, Lindeman JH, Klautz A, Driessen A, Klautz RJM, et al. Normal and abnormal development of the aortic valve and ascending aortic wall: a comprehensive overview of the embryology and pathology of the bicuspid aortic valve. Ann Cardiothorac Surg. 2022;11:380–8. 10.21037/acs-2021-bav-14 PMC9357963 35958528

[3] Hiratzka LF, Creager MA, Isselbacher EM, Svensson LG, Nishimura RA, Bonow RO (2016). Surgery for aortic dilatation in patients with bicuspid aortic valves: a statement of clarification from the American College of Cardiology/American Heart Association Task Force on Clinical Practice Guidelines. Circulation.

[4] Grewal N, Girdauskas E, deRuiter MC, Goumans MJ, Lindeman JH, Disha K, et al. The effects of hemodynamics on the inner layers of the aortic wall in patients with a bicuspid aortic valve. Integr Mol Med. 2017;4:1–7.

[5] Grewal N, Girdauskas E, DeRuiter M, Goumans MJ, Poelmann RE, Klautz RJM (2019). The role of hemodynamics in bicuspid aortopathy: a histopathologic study. Cardiovasc Pathol.

[6] Grewal N, Franken R, Mulder BJM, Goumans MJ, Lindeman JHN, Jongbloed MRM (2016). Histopathology of aortic complications in bicuspid aortic valve versus Marfan syndrome: relevance for therapy?. Heart Vessels.

[7] Grewal N, Gittenberger-de Groot AC, von der Thousen J, Wisse LJ, Bartelings MM, DeRuiter MC, et al. The development of the ascending aortic wall in tricuspid and bicuspid aortic valve: a process from maturation to degeneration. J Clin Med. 2020;9:908. 10.3390/jcm9040908 PMC7230962 32225051

[8] Li Y, Lui KO, Zhou B (2018). Reassessing endothelial-to-mesenchymal transition in cardiovascular diseases. Nat Rev Cardiol.

[9] Franken R, den Hartog AW, de Waard V, Engele L, Radonic T, Lutter R (2013). Circulating transforming growth factor-β as a prognostic biomarker in Marfan syndrome. Int J Cardiol.

[10] Junco-Vicente A, Del Río-García Á, Martín M, Rodríguez I. Update in biomolecular and genetic bases of bicuspid aortopathy. Int J Mol Sci. 2021;22:5694. 10.3390/ijms22115694 PMC8198265 34071740

[11] Alvandi Z, Bischoff J (2021). Endothelial-mesenchymal transition in cardiovascular disease. Arterioscler Thromb Vasc Biol.

